# Breast Metastasis From Uterine Leiomyosarcoma: A Case Report

**DOI:** 10.7759/cureus.109908

**Published:** 2026-05-30

**Authors:** Evangelos P Solakis, Nikolaos P Tasis, Christos Kalyvopoulos, Athina Kapsokoulou, Christos Batsis

**Affiliations:** 1 Department of Breast Surgery, 401 General Military Hospital of Athens, Athens, GRC; 2 Department of Surgical Oncology, Saint Savvas General Anti-Cancer Hospital of Athens, Athens, GRC; 3 First Department of Breast Surgery, Saint Savvas General Anti-Cancer Hospital of Athens, Athens, GRC; 4 Department of Obstetrics and Gynaecology, Second Health Care Center of Peristeri, Athens, GRC; 5 Second Department of Breast Surgery, Saint Savvas General Anti-Cancer Hospital of Athens, Athens, GRC

**Keywords:** breast cancer, breast metastasis, case report, leiomyosarcoma, metastatic breast tumor, metastatic sarcoma, sarcoma, uterine leiomyosarcoma

## Abstract

Breast metastases from extramammary malignancies are uncommon. Among these, metastatic spread from uterine leiomyosarcoma is exceptionally rare. Uterine leiomyosarcoma is an aggressive mesenchymal neoplasm characterized by early hematogenous dissemination, most commonly to the lungs, liver, peritoneum, and bone. Breast involvement is distinctly unusual and may mimic primary breast carcinoma both clinically and radiologically. We report the case of a 76-year-old woman who presented with a painless enlarging mass in the upper outer quadrant of the left breast three months after hysterectomy with bilateral salpingo-oophorectomy for high-grade uterine leiomyosarcoma. Fine-needle aspiration suggested metastatic mesenchymal neoplasm, and wide local excision confirmed metastatic leiomyosarcoma. This case highlights the importance of considering metastatic disease in patients with a history of uterine sarcoma who develop new breast lesions.

## Introduction

Metastatic involvement of the breast by non-mammary malignancies is rare, representing approximately 0.5-2% of all breast malignancies. The most common sources include melanoma, lymphoma, lung carcinoma, ovarian carcinoma, and sarcomas [1,2]. Among metastatic sarcomas to the breast, uterine leiomyosarcoma is one of the rarest reported entities [3].

Uterine leiomyosarcoma accounts for approximately 1-2% of uterine malignancies and is the most common malignant mesenchymal tumor of the uterus. It is known for aggressive behavior, high recurrence rates, and a tendency for distant hematogenous spread. The lung is the most frequent metastatic site, followed by the peritoneum, bone, liver, soft tissues, and, rarely, the breast [4].

Because breast metastases may resemble benign lesions or primary breast carcinoma on imaging, diagnosis can be challenging. Recognition is clinically important, as treatment strategies differ significantly from those for primary breast cancer. We present a rare case of isolated breast metastasis from uterine leiomyosarcoma diagnosed shortly after hysterectomy, along with a review of the literature.

## Case presentation

A 76-year-old postmenopausal woman was referred to a breast clinic because of a progressively enlarging palpable mass in the upper outer quadrant of the left breast. The lesion had been present for several months but had recently increased in size. It was painless and non-tender, with no associated skin changes, nipple discharge, or constitutional symptoms.

Her medical history was significant for hypertension, dyslipidemia, hypothyroidism, and depression, all controlled with medication. Previous surgical history included an appendectomy during childhood. She had two prior uncomplicated vaginal deliveries and menopause at the age of 51 years. There was no family history of breast or gynecologic malignancy.

**Figure 1:**
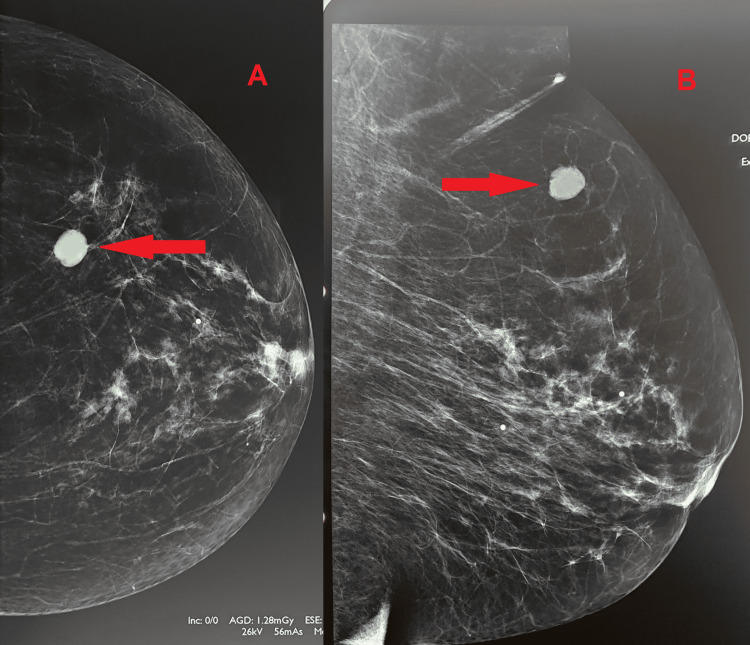
Mammography findings. (A) Craniocaudal view. A well-circumscribed, round, hyperdense mass (red arrow) is identified in the upper outer quadrant. (B) Mediolateral oblique view of the same breast confirms the presence of the lesion (red arrow) in the superior third, without evidence of architectural distortion or skin thickening. Breast parenchymal composition is characterized as American College of Radiology category B.

Several months earlier, the patient had presented with postmenopausal vaginal bleeding. Imaging investigations demonstrated uterine enlargement with heterogeneous changes involving the endometrium and myometrium, suspicious for neoplastic infiltration, in addition to coexisting leiomyomatous disease. At that time, comprehensive preoperative evaluation revealed no evidence of distant metastatic disease apart from the known uterine tumor. During the preoperative assessment, mammography (Figure 1) identified a small nodular lesion in the left breast with benign-appearing radiologic characteristics, including circumscribed margins and oval shape, and no suspicious calcifications or spiculations.

She subsequently underwent a total abdominal hysterectomy with bilateral salpingo-oophorectomy. Histopathological examination revealed a uterine leiomyosarcoma measuring 18 cm in greatest dimension. Microscopy demonstrated marked nuclear atypia, increased mitotic activity (>10 mitotic figures per 10 high-power fields), and areas of coagulative tumor necrosis, consistent with high-grade aggressive behavior. Adjuvant chemotherapy and radiotherapy were recommended following surgery; however, the patient declined further oncologic treatment.

**Figure 2:**
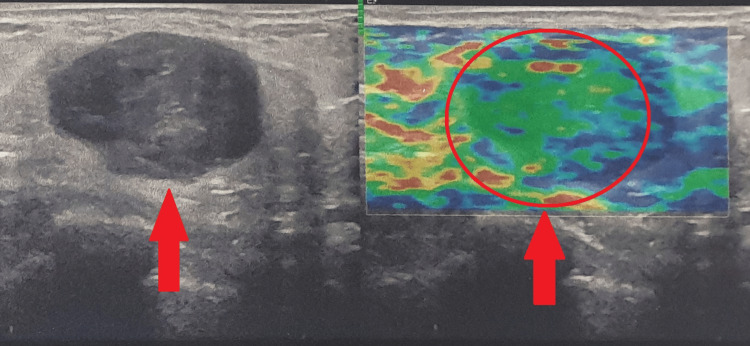
Ultrasound findings. Grayscale ultrasound image demonstrates a large, heterogeneous, predominantly hypoechoic mass with mildly irregular and smooth margins and possibly areas of internal necrosis (left red arrow). Corresponding real-time strain elastography map of the same region with increased stiffness (right red arrow area).

Two months after the hysterectomy, the patient presented to the outpatient breast clinic because of interval enlargement of the known breast lesion, prompting repeat clinical and radiologic assessment. Physical examination confirmed a firm, mobile mass in the left breast without palpable axillary lymphadenopathy. Targeted breast ultrasonography (Figure 2) demonstrated a 3.7 cm solid lobulated hypoechoic lesion with well-defined margins, increased internal vascularity, and increased stiffness on elastographic evaluation.

Fine-needle aspiration cytology was performed because of the lesion’s rapid growth and the patient’s recent history of uterine malignancy. Cytologic examination showed a malignant spindle-cell mesenchymal neoplasm, findings considered highly suggestive of metastatic leiomyosarcoma. Systemic staging included chest radiography, bone scintigraphy, and serum tumor marker evaluation (carcinoembryonic antigen, cancer antigen 125, and cancer antigen 15-3), all of which were within normal limits and showed no evidence of additional metastatic disease. Contrast-enhanced computed tomography of the thorax or positron emission tomography/computed tomography was not performed because these modalities were not included in the institutional protocol at the immediate postoperative time following hysterectomy, and a complete preoperative evaluation was undertaken at that time.

**Figure 3:**
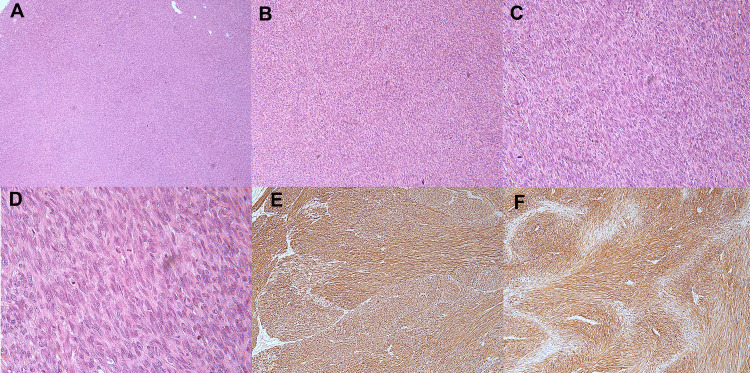
Immunohistochemical findings of the breast lesion. (A) Low-power view (hematoxylin and eosin (H&E), ×4) showing a densely cellular spindle cell neoplasm composed of intersecting fascicles and bundles. (B) Intermediate-power view (H&E, ×10) demonstrating fascicular growth of atypical spindle cells with eosinophilic cytoplasm and infiltrative architecture. (C) High-power view (H&E, ×20) revealing malignant smooth muscle cells with elongated hyperchromatic nuclei, moderate pleomorphism, and increased cellularity. (D) High-power view (H&E, ×40) showing marked cytologic atypia, nuclear pleomorphism, and increased mitotic activity, consistent with leiomyosarcoma. (E) Desmin immunohistochemical staining demonstrating diffuse strong cytoplasmic positivity in tumor cells, supporting smooth muscle differentiation. (F) h-Caldesmon immunohistochemical staining showing diffuse strong cytoplasmic expression in neoplastic spindle cells, confirming smooth muscle lineage.

Following multidisciplinary discussion, the breast lesion was considered an isolated metastatic focus, as no additional sites of metastatic disease were identified on systemic evaluation. Therefore, breast-conserving surgical excision was performed with curative intent in the setting of presumed oligometastatic disease and to achieve local disease control. The patient underwent breast-conserving surgical excision under general anesthesia. The resected specimen measured 4.5 × 3 × 2.1 cm. Histopathological examination confirmed metastatic leiomyosarcoma involving the breast parenchyma, graded as French Federation of Cancer Centers Sarcoma Group grade 2 [5]. Tumor cells showed positive immunoreactivity for desmin and h-caldesmon, supporting smooth muscle differentiation, while estrogen receptor, progesterone receptor, and S100 were negative (Figure 3). Surgical margins were free of tumor.

Following a multidisciplinary review, adjuvant systemic therapy was again recommended because of the metastatic nature of the disease. However, the patient declined further oncologic treatment after detailed counseling regarding potential benefits and risks. Consequently, she was managed with close clinical and radiologic surveillance. At the six-month postoperative follow-up, the patient remained free of local recurrence or evidence of additional metastatic disease.

## Discussion

Metastatic involvement of the breast by non-mammary malignancies is an uncommon clinical entity, accounting for approximately 0.5-2% of all malignant breast tumors in surgical and pathological series. These lesions are clinically important because their management and prognosis differ substantially from those of primary breast carcinoma. Among metastatic tumors to the breast, the most common primary sites include melanoma, lung, ovary, gastrointestinal tract, and hematologic malignancies, whereas sarcomatous metastases are distinctly rare [1,2]. Within this subgroup, uterine leiomyosarcoma represents an uncommon source [4].

Uterine leiomyosarcoma is a rare but highly aggressive mesenchymal malignancy, comprising approximately 1-2% of uterine cancers and representing the most frequent uterine sarcoma subtype. It is characterized by early hematogenous dissemination, high recurrence rates, and poor long-term survival. The most common metastatic sites are the lungs, peritoneum, liver, and bone. In a pooled clinicopathologic analysis of 130 patients with metastatic uterine leiomyosarcoma, Bartosch et al. reported lung metastases in 67.7% of cases, followed by cranial/intracranial (16.2%), soft tissue/skin (15.3%), and bone (13.8%) involvement; breast metastases were documented only rarely, underscoring the nature of this presentation [3].

Breast metastasis from uterine leiomyosarcoma has been described only in isolated case reports and small case series [4,6-12]. Reported intervals between diagnosis of the uterine primary and breast involvement vary widely, from synchronous disease to metastasis occurring more than a decade after hysterectomy. This broad temporal spectrum reflects the unpredictable biological behavior of uterine leiomyosarcoma and emphasizes the need for prolonged surveillance [4]. Our patient developed a breast metastasis only three months after hysterectomy, suggesting biologically aggressive disease with early systemic spread despite negative conventional staging at presentation. Similar early presentations have been reported, although delayed recurrences appear more common in published literature [3].

Clinically, metastatic lesions to the breast usually present as painless, rapidly enlarging, mobile masses, often without skin or nipple changes [12]. Axillary nodal involvement is less common than in primary breast carcinoma because dissemination is predominantly hematogenous rather than lymphatic. Radiologically, these lesions frequently appear as well-circumscribed, round or oval masses without spiculation, architectural distortion, or suspicious microcalcifications. On ultrasound, they are often hypoechoic and hypervascular [7,11]. Consequently, metastatic lesions may mimic benign entities such as fibroadenoma or circumscribed primary carcinomas [10]. In the present case, the lesion had initially benign mammographic features before demonstrating interval growth, highlighting the importance of clinical vigilance and repeat assessment in patients with known malignancy.

Histopathological confirmation is essential, as imaging alone cannot reliably distinguish metastatic disease from a primary breast neoplasm. Fine-needle aspiration, core biopsy, or excisional biopsy may establish the diagnosis. Cytologically, metastatic leiomyosarcoma typically demonstrates spindle-cell or pleomorphic mesenchymal cells with hyperchromatic nuclei, eosinophilic cytoplasm, and variable mitotic activity [4,7,11]. Immunohistochemistry is crucial for lineage confirmation, with positivity for smooth muscle markers such as desmin, smooth muscle actin, h-caldesmon, and vimentin, while epithelial markers (cytokeratins, GATA3), hormone receptors, and other lineage markers are usually negative. This immunophenotype assists in excluding metaplastic spindle-cell carcinoma, malignant phyllodes tumor, primary breast sarcoma, fibromatosis, and melanoma [4].

Recognition of metastatic disease has significant therapeutic implications. Unlike primary breast carcinoma, routine mastectomy, sentinel lymph node biopsy, and breast cancer-specific systemic regimens are generally not indicated unless required for palliation or local control. Management should instead be guided by the biology and extent of the primary malignancy. For isolated breast metastasis, local excision may be appropriate to achieve diagnosis and symptom control [2,9]. In disseminated disease, systemic therapy directed toward uterine leiomyosarcoma is preferred, including anthracycline-based chemotherapy, gemcitabine/docetaxel combinations, trabectedin, pazopanib, or enrollment in clinical trials, depending on prior therapy and performance status. Surgical resection of limited metastatic deposits may offer benefit in selected patients. In the study by Bartosch et al., metastasectomy was the only intervention significantly associated with improved post-metastatic survival on multivariable analysis [3].

Our case contributes to the limited literature on breast metastasis from uterine leiomyosarcoma and reinforces several practical messages: first, any new breast mass in a patient with prior sarcoma warrants tissue diagnosis; second, benign-appearing imaging does not exclude metastasis; third, correlation with oncologic history and pathology review is essential; and, finally, treatment should be individualized within a multidisciplinary sarcoma framework.

## Conclusions

Breast metastasis from uterine leiomyosarcoma is an exceptionally rare event that may mimic primary breast neoplasia or even benign lesions on imaging. A high index of suspicion is required when evaluating a new breast mass in any patient with a history of uterine sarcoma, regardless of the interval since primary diagnosis. Histopathological confirmation is essential to establish the diagnosis and avoid unnecessary mastectomy or inappropriate breast cancer-directed treatment. Multidisciplinary individualized management remains crucial.
